# Artificial intelligence for cancer screening and surveillance

**DOI:** 10.1016/j.esmorw.2024.100046

**Published:** 2024-07-10

**Authors:** F. Gentile, N. Malara

**Affiliations:** 1 Department of Experimental and Clinical Medicine, University Magna Graecia, Catanzaro, Italy; 2 Nanotechnology Research Center, University Magna Graecia, Catanzaro, Italy; 3 Department of Health Sciences, University Magna Graecia, Catanzaro, Italy

**Keywords:** prediction models, AI models in health care, AI-powered personalized medicine, data integrity, prediction model validation, liquid biopsy

## Abstract

Investing in cancer prevention can be cost-effective. However, this requires significant changes both inside and outside the health care system. The core of the preventive strategy is the assignment of an individual risk level of developing cancer. Artificial intelligence (AI), which has emerged as a tool to reduce errors and confusion in data collection and analysis, has helped accelerate recent advances in identifying circulating markers to generate predictive methods. With predictive models applied to increasingly less invasive and repeatable analytic tests, the risk is no longer assigned but profiled directly on the individual over time. On this basis, the probability of early cancer diagnosis is increased and at the same time, proactive preventive medicine transits from offering lifestyle recommendations to guiding specific treatments to reduce the risk. Despite these promises, AI-based predictive models also present challenges in clinical implementation. Addressing these challenges is crucial to minimizing the future burdens associated with fighting cancer.

## Highlights

• The need for fit-for-primary prevention purpose AI is growing in oncology.
• AI and noninvasive detection procedures are partnering to assess cancer prevention.
• Screening tools eligible for risk management must be validated.
• Robust methods for prediction model validation are necessary.

## Introduction

Historically perceived as an unattainable goal, prevention in oncology is now gaining relevance through the integration of precision medicine with digital techniques.^1^^,^^2^ This synergy has revolutionized prevention in oncology with the formulation, development, and establishment of screening programs ranging from proactive assessment of oncological risks to early diagnosis platforms.^3^ We distinguish three levels of tumour prevention: primary, secondary, and tertiary. Primary prevention focuses on avoiding the disease completely, so interventions are carried out to reduce the risk before an illness occurs. Secondary prevention includes conducting screenings to provide an early diagnosis for the patient. Tertiary prevention focuses on reducing the risk of new lesions, referred to as relapses, and potential metastases following cancer treatment. Liquid biopsy (LB) has an important role in tertiary prevention and is rapidly growing in primary prevention and secondary areas. LB is a noninvasive test (often a blood test) that detects signs of cancerous tumours, including molecular and cellular markers.^3^ Initially developed for oncology, this approach was later expanded to include other diseases.^4^ The growing range of biomarkers offered by LB may improve cancer prevention, which has historically been hindered by the absence of established and verified biomarkers. In LB, biomarkers are designed, identified, and strategically chosen to accurately predict an individual’s level of biological risk in real-time and to evaluate the effectiveness of strategies implemented to mitigate this risk.^5^ LB has significantly improved the management of patients with cancer through the synchronic monitoring of cancer development and progression and effectively redefined the concept of cancer risk, previously understood as a combination of positive and negative lifestyle factors, within a framework of biosafety in which biological risk is quantified through the analysis of biomarkers related to cell damage.^6^ Over the past decade, the clinical use of LB, initially aimed at reducing or controlling morbidity in established cancers (tertiary prevention), has expanded to screen patients for early diagnosis or secondary prevention and more recently, in primary prevention. In this field, LB aims to prevent the clinical manifestation of cancer through interventions that detect risk and implement control measures to reduce it and prevent the disease.^7^ While LB shows promise in establishing itself as a standard in clinical practice, it requires greater effort to achieve this status. Preventive medicine, and therefore LB, has long faced a series of seemingly insurmountable challenges, in particular:

The excessively long validation time of biomarkers. This is mainly because evaluating the expected effects on cancer as a definitive endpoint could take decades.^8^

The characterization of patient populations with subclinical disease, which are generally underrepresented in clinical trials.^9^

Not least are the difficulties associated with communication between doctors and potential candidates for preventive therapy, which include estimating, calculating, and explaining their risks, as well as illustrating the potential benefits of available options.^10^

In recent years, the growing adoption of artificial intelligence (AI)-based models has enhanced the ability to tackle these challenges in preventive medicine.

**Figure 1:**
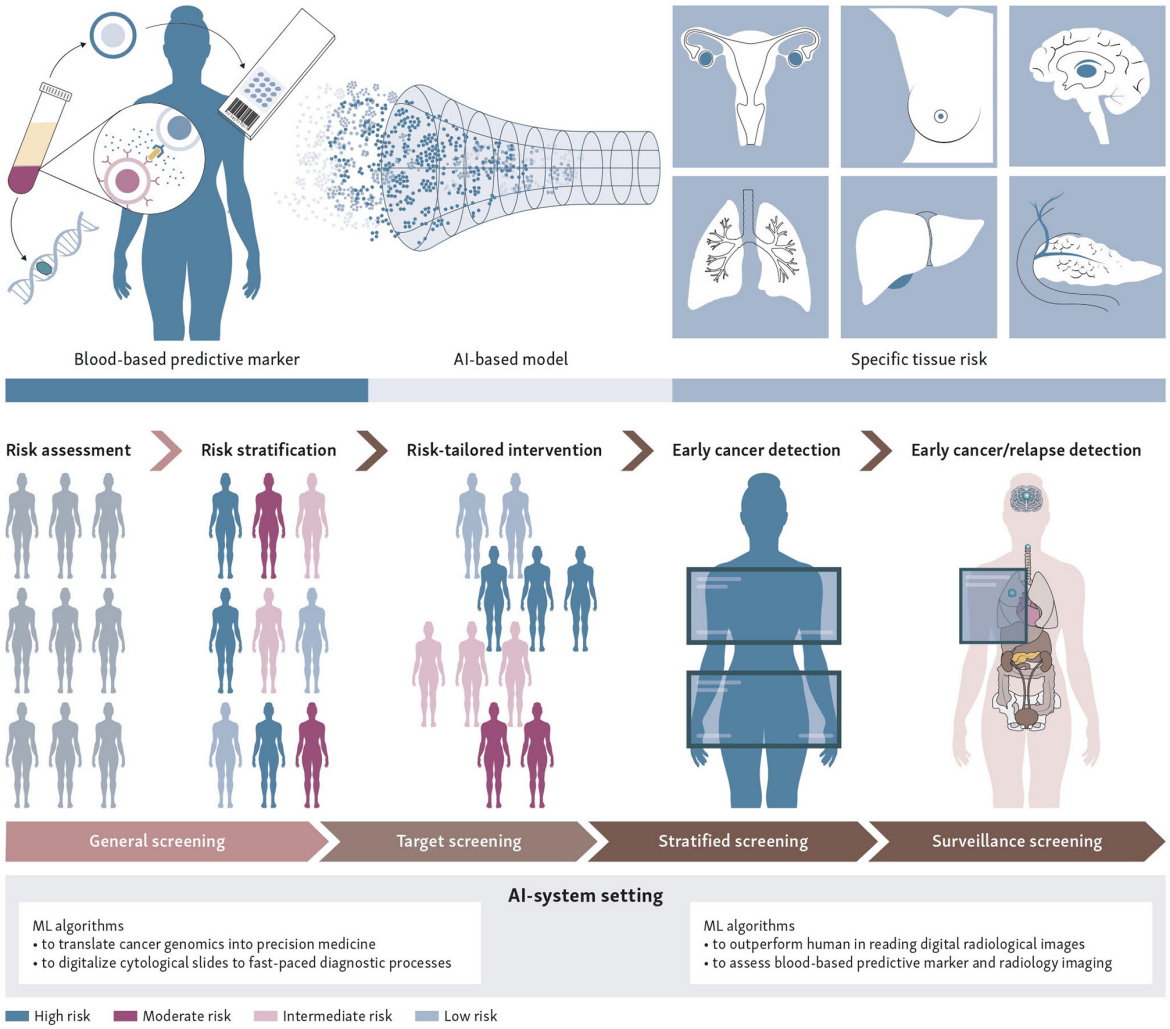
**Personalized risk assessment and artificial intelligence (AI) applications addressed in real-world settings for screening and cancer surveillance.** Blood-based personalized assessments using genomic alterations and machine learning (ML) algorithms shift patients from general to targeted cancer screening. These screenings offer insights for informed decision making and potential risk-reducing treatments. High-risk individuals undergo combined blood and radiological monitoring, transitioning from targeted to stratified screening. This approach also applies to surveillance screening for cancer survivors, integrating blood-based markers with radiology. The type of AI used in these studies is ML algorithms to answer the predictive question of the test. The stratification and classification of data created by supervised or unsupervised ML systems form the basis of predictive medicine. The validation process in current studies is mainly limited to verifying the predictive efficiency of the model using data from external datasets.

These aforementioned long-standing problems are now being addressed more effectively with the use of AI applications (in particular, AI debuted as a method to systematically collect and process individual data using specialized algorithms).^11^ Examples of this are (i) the use of predictive models in retrospective studies to shorten validation times; (ii) the use of neural networks (NNs) for the characterization and identification of subclinical cases along with risk assessment; and (iii) the application of AI for enhancing patient interface, helping to bridge distances and overcome communication barriers. These advances have collectively initiated a new cycle of screening options in cancer prevention, including three main programs, general population screening, targeted screening, and stratified screening as shown in Figure 1.

Each of these screening programs involves proactive approaches to identify an increasing degree of health problems in various segments of the population. General population screening invites specific demographic groups, defined by criteria such as age or gender, to participate in screenings that facilitate informed health decisions^12^ and drug recommendations to reduce risk. Targeted screening focuses on populations at risk due to genetic variants or pre-existing health conditions,^13^ while stratified screening customizes the frequency and type of testing based on the individualized risk level.^14^ The stratified screening program represents the latest frontier of primary prevention by identifying the needs which then divert patients towards secondary prevention aimed at diagnosis. This enables early treatment and a faster return to baseline health values.

This overview highlights the experiments, methods, and statistical approaches used in studies based on AI-based screening models and outlines new barriers that must be overcome to advance this promising field and achieve desperately needed reductions in the incidence of cancer.

## AI-based primary cancer prevention

Over the past five years, only a limited number of AI-based risk assessment studies have focused on identifying biomarkers that effectively stratify cancer risk within the general population. Table 1 shows a selection of experiments using AI in various aspects of preventive medicine, in particular in the screening and early diagnosis of different types of cancer. Each of these studies represents a significant advance in the application of AI in medical diagnostics, showing the potential of NNs and deep learning (DL) approaches to improve the accuracy and efficiency of disease detection and screening. These advanced methods utilize quantitative image analysis tools that produce detailed information. These data are processed by NNs and incorporated into a dataset to categorize the population, including individuals without a family history or clinical signs of disease, into different risk classes. In this context, the most frequently used type of machine learning (ML) relies on algorithms derived from deep neural networks (DNNs) or convolutional neural networks (CNNs). Table 1 demonstrates the versatility of AI applications in the health care sector, illustrating a broad spectrum of diseases treated—from colon cancer to cervical neoplasia—and a diverse array of applications, from predicting diseases using laboratory data to detecting cancer noninvasively through genetic profiling. Different methodologies, including DNNs, semisupervised learning, and principal component analysis, highlight the dynamic nature of AI applications in health care. Validation of these systems is typically carried out through orthogonal validation tests or by applying the algorithm to a dataset within a different clinical context. These initial studies typically involve a relatively small number of patients but generate a relatively large amount of data from those participants. The validation process of the predictive model in these studies is more complex, as it must be structured through a series of steps. These steps are designed to validate both the analytical and predictive aspects of the model, both individually and collectively. To ensure test repeatability and effectively interpret the individual data collected, biomarkers must be easily obtainable via noninvasive methods. This ensures that the analysis can be repeated without affecting the individual’s baseline health status. Recent studies examining fragments of cell-free DNA (cf-DNA) in plasma demonstrate how it is possible to measure the degree of cancer risk from a blood sample.^5^ The AI system used is an ML framework that analyses the frequency of mutations in genomic regions of circulating DNA, which are commonly altered in cancer.^5^ This system considers individual variability by distinguishing probable somatic mutations from the background of general genomic changes. As a result, it allows cancer risk assessment to be tailored to the individual. A retrospective study for noninvasive early detection of lung cancer used 255 genes to determine detection rates of circulating tumour DNA in patients (study 1). The Lung-CLiP model, based on semisupervised learning, was used. The validation included an orthogonal evaluation of 15 white blood cell cfDNA mutations in circulating tumour DNA and compared the results with genomic features. The cf-DNA blood biomarker for noninvasive cancer detection is also being studied, as part of the National (USA) Lung Screening Trial, an innovative approach using whole-genome sequencing data from 2511 individuals. The goal was to develop a noninvasive method for cancer diagnosis, a significant breakthrough in cancer diagnostics. The use of an ML model based on principal component analysis of fragmentation patterns is a sophisticated approach, reflecting the complex nature of genomic data analysis. The validation involving an additional cohort of individuals shows the study’s commitment to in-depth and comprehensive scientific investigation (study 2).

**Table 1:** A selection of experiments utilizing AI in various aspects of preventive medicine, particularly in the screening and early detection of different types of cancer and neoplasms

Screening type	RCT	Dataset	Aim	Tool	Data	Algorithm	Validation	Reference
1. Lung cancer detection	Retrospective study (missing ID)	255 genes	Improve early detection	Lung-CLiP model, semisupervised learning	Genomic features	3NN, naive Bayes, logistic regression, and decision tree	Orthogonal validation of 15 WBC cfDNA mutations and 46 lung cancer cases	^13^
2. Cancer detection	National Lung Screening Trial	Whole-genome sequencing data from 2511 individuals	Improve early detection	ML model using genome-wide mutational profiles	Whole-genome sequencing data	Logistic regression model with LASSO penalty	Evaluation of an additional cohort of individuals from lung cancer screening programs	^5^
3. Cervical intraepithelial neoplasia	Multicentre (ID ChiCTR2000034121)	188 542 cervical cell images	Improve early detection	Automation-assisted and manual cytology reading in cervical screenings	Images	DL algorithm with CNN	Choosing the model with the highest *F*_1_-score	^18^
4. Cervical precancer	Multicentre (missing ID)	60 patients	Improve early detection	Diagnostic method optimized by AI	Colposcopy images	CNN	External dataset	^19^
5. Colon cancer	Multicentre (ID NCT04422548)	112 199 colonoscopy images	Improve polyp detection	Compare AI-assisted colonoscopy with conventional colonoscopy	Images/video	Deep CNN	Using an independent dataset of 40 000 colonoscopy images	^20^
6. Colorectal cancer surveillance	Multicentre (ID DRKS00023157)	96 patients with Lynch syndrome	Improve early detection	Compare the AI-assisted group with the unaided group	Images	CNNs	Only an exploratory study. Data need to be validated in a prospective randomized multicentre study	^21^
7. Oesophageal squamous cell carcinoma	Multicentre (ID ChiCTR2100052116)	5934 patients	Improve early detection	Compare the AI-assisted group with the unaided group	Images	ANN	Paywalled scientific article	^22^
8. Skin neoplasm	Multicentre (missing ID)	576 cases	Improve early detection	Compare the AI-assisted group with the unaided group	Images	CNN	Conducted only in Asians, mostly with Fitzpatrick skin phototypes III and IV	^23^
9. Hepatocellular carcinoma	Multicentre (missing ID)	504 patients	Establish ALBI grade	An ANN model to compare the prognostic performance	BCLC stage, ALBI grade	ANN	ALBI grade, Childs-Turcotte-Pugh score, BCLC stage, maximum tumour size, intrahepatic tumour number, intrahepatic tumour location, and plasma alpha-fetoprotein (≤200 ng/dl or >200 ng/dl)	^24^
10. Barrett’s carcinoma	Multicentre (missing ID)	1020 images	Accuracy of T stage of Barrett’s carcinoma	Compare the AI-assisted group with the unaided group	Images	AI system based on endoscopic images	Paywalled scientific article	^25^
11. Diagnostic disease prediction	No trial	5145 cases and 326 686 laboratory test	Improve early detection	Compare the DL model with two ML models	Patients laboratory data	DNN model + ML (XGBoost and LightGBM)	Stratified fivefold cross-validation for DL and ML models and validationloss for the Ensemble model (DL + two ML)	^12^
12. Lung cancer	Multicentre RCT National Lung Screening Trial and NELSON trial	888 scans	Improve early detection	AI-based volumetric segmentation algorithms	Images	NN	Tested on the same 50 anonymized scans and evaluated using the same procedure	^26^
13. Breast cancer	Multicentre RCT (The Avon-ACRIN American College of Radiology multicentre study)	1 163 147 women	Improve early detection	Software to calculate density combined with AI cancer detection system	Images	Deep CNN	2222 women diagnosed with interval cancer within 20 months of a negative mammogram	^27^
14. Breast 2cancer	Large-scale retrospective study (missing ID)	3746 women	Improve early detection	State-of-the-art ML tools for image classification	Images	Deep CNNs	External large-scale collection of >1 million images	^2^

Each of these studies represents a significant advancement in the application of AI in medical diagnostics, showcasing the potential of DL and NNs in enhancing the accuracy and efficiency of disease detection and screening. The diverse range of diseases covered, from colon cancer to cervical neoplasia, and the diverse range of applications, from predicting diseases using laboratory data to noninvasive cancer detection using genetic profiling, highlights the versatility of AI applications in health care. The diverse methodologies, including DNN, semisupervised learning, and principal component analysis, highlight the dynamic nature of AI applications in health care.

3NN, 3 nearest neighbours; AI, artificial intelligence; ALBI, albumin–bilirubin; ANN, artificial neural network; BCLC, Barcelona Clinic Liver Cancer; cfDNA, circulating-free DNA; CNN, convolutional neural network; DL, deep learning; DNN, deep neural network; LASSO, Least Absolute Shrinkage and Selection Operator; LightGBM, Light Gradient-Boosting Machine; ML, machine learning; NN, neural network; RCT, randomized controlled trial; WBC, white blood cell; XGBoost, Extreme Gradient Boosting.

The limitation of the studies of individual variability based on the detection of somatic mutation is that it does not fill the prerequisite of a tissue-specific reference. Somatic mutations of some genes are common to many tumours arising in different body areas (i.e. *RAS* mutation found in a blood sample could indicate the presence of tumour but it is not specific for the site because colon, lung, and pancreatic cancer are highly linked to *RAS* patterns). To be effective, prevention must be able to focus on those specific organs that have a critical oncological risk. Therefore research efforts in this field are aimed at identifying tissue-specific markers, focusing on the surveillance of high-risk activities. The application of AI as digital cytological or histological pathology opens a further page of proactive targeted screening as in the study on Cervical Intraepithelial Neoplasia (study 3, 4). In this multicentre randomized controlled trial (RCT), 188 542 cervical cell images were analysed to evaluate the performance of AI-assisted cytology. The study used a DL algorithm with CNN and compared automation-assisted with manual cytology readings. The model chosen for use had the highest *F*_1_-score, which was a key statistical measure in this study. The limitation of this study is the type of sampling, which regarded some women as invasive and not suitable for a screening program to be repeated over time.

Considering that the blood sample is better tolerated for a screening analysis and that the comfort of a cytological analysis could be crucial for an accurate early cancer diagnosis, some researchers have focused on improving blood-based cytological evaluation. One of the protocols in the field of LB focused on this issue is the Charactex protocol allowing to obtain cytological preparations enriched for nonhaematological cells from peripheral blood.^15^ A recent study,^7^ combining the NN model and blood-based cell specimens, shows the assessment of tissue-related cancer risk through the detection of proliferating atypical epithelial cells. The analysis of antigenic expression of epithelial cells fixes the problem of tissue site identification. This type of approach directs screening towards targeted surveillance. This information opens to surveillance activities such as imaging, nutraceutical approaches, and pharmacological treatments, for the effective management of high risk. Furthermore, the possibility of repeating the blood examination guarantees continuous and real-time monitoring of the treatment effects *on that tissue in that person at that time* according to the emerging precision preventive medicine approach.

## Secondary and tertiary cancer prevention based on AI

Most research on AI-based screening models includes multicentre retrospective studies, primarily on the analysis of clinically identified lesions with radiological imaging. Retrospective studies, which use existing data recorded for nonresearch purposes, improve predictive assessments and allow the safety and effectiveness of AI to be tested before its potential application. Most of the studies conducted evaluate AI diagnostic interpretation on radiological or endoscopic images using supervised or unsupervised ML models. The performance of these models is compared with routine interpretations made by specialists.^12^ Many studies use—to a large extent—ML algorithms based on artificial neural networks (ANNs) applied to radiological images that undergo a segmentation process and are validated against an external dataset (Table 1, studies 2-11). These methods and statistical approaches are used in colon cancer screening studies to improve the polyp detection rate. The multicentre study RCT compared AI-assisted colonoscopy with conventional methods using a deep CNN in a test campaign of 112 199 colonoscopy cases. Validation was carried out with an independent dataset of 40 000 colonoscopy images (study 5). In addition, the Colorectal Cancer Surveillance study, a multicentre RCT with 96 patients with Lynch syndrome, aimed to investigate the diagnostic performance of AI in colorectal cancer surveillance. The method involved comparing an AI-assisted group with an unaided group, using CNN. This study was exploratory, and the data needed further validation (study 6) as the multicentre RCT on oesophagal squamous cell carcinoma involved 5934 patients divided into an AI-first group and a control group. The aim was to improve early diagnosis using an ANN, with the study focusing on image analysis (study 7).

AI has also been used in the detection of skin cancers. This study, also a multicentre RCT, used 576 cases of suspicious lesions (Fitzpatrick skin types I-IV) to improve the detection of skin malignancies. CNNs were used in this comparison between AI-assisted and unaided groups. Validation was conducted only in Asians, primarily those with Fitzpatrick skin types I-IV (study 8). Imaging and laboratory data are combined in the RCT using an ANN on a dataset with 504 hepatocellular carcinoma cases, including various clinical parameters such as Barcelona Clinic Liver Cancer (BCLC) stage, albumin–bilirubin grade, maximum tumour size, and computed tomography images. The ANN model was compared with existing prognostic systems such as Childs-Turcotte-Pugh score and Barcelona Clinic Liver Cancer staging (study 9). A multicentre RCT on the imaging of precancerous lesions, such as Barrett’s oesophagus, involving 1020 patients aimed to study the accuracy of an AI system based on endoscopic images. The comparison was made between AI-assisted groups and non-AI-assisted groups (study 10).

These studies demonstrate how ML—based on NNs—can stratify the population using the degree of disease and/or risk as a criterion. Here the clinician contributes to the construction of the predictive model by supervising the learning and validation phases of the algorithm. The validation process verifies how well the predictive model works on unstructured data: it verifies the functioning of the model in unsupervised mode using external data sets as input, different from those used in the learning phase.

In this context, the literature also includes a diagnostic disease prediction: This study, not associated with a trial, analysed 5145 cases encompassing 326 686 laboratory test results and electronic health records. The aim was to build a new optimized ensemble model by blending DL with ML techniques, specifically, DNN models combined with Extreme Gradient Boosting (XGBoost) and Light Gradient-Boosting Machine (LightGBM). The validation was conducted through stratified fivefold cross-validation for DL and ML models, focusing on patients’ laboratory data for diagnosis (study 11).

From a clinical perspective, AI-based screening in clinical practice for lung cancer has also shown a measurable increase in cancer detection rates with consistent results even when AI is applied in daily screening routines. In particular, the clinical benefits observed were a significant increase of 5%-13% in the early diagnosis of mostly invasive and small tumours and minimal impact on further false-positive recalls. In the Lung Cancer Screening study (study 12), part of the National Lung Screening Trial used AI-based volumetric segmentation algorithms on 888 scans. The aim was to detect and characterize pulmonary nodules. The AI algorithms were tested on 50 anonymized scans and evaluated for their effectiveness in lung cancer screening. The AI-based models used to screen patients with breast cancer (study 13, 14) included various types of risk prediction by incorporating several factors, including demographic, clinical and genetic parameters, as well as imaging. Important predictive models integrate radiological data with aspects such as family history, breast density, and genetic markers. These studies make use of ML based on ANNs—applied to both data obtained from segmentation of radiological images and molecular and clinical data, as in the breast cancer, a large-scale study that is part of the Avon-ACRIN American Study College of Radiology Imaging Network, and involved 1 163 147 women from the Dutch screening program. The aim was to find cancer early to reduce mortality. The study used software for calculating density combined with AI methods, particularly deep CNN. The validation involved 2222 women diagnosed with interval cancer within 20 months of screening, and in the large-scale retrospective study, this research involved 3746 women. The goal was to demonstrate the clinical benefits of state-of-the-art ML tools for image analysis, using deep CNNs. Validation was carried out on a diverse, large-scale collection of mammographic images. Although the potential of AI to transform preventative medicine is encouraging, the successful application of retrospective findings in real-world clinical practice depends on the representativeness of the data. This is important to ensure that AI works effectively in real-world scenarios. Consequently, although AI-based clinical assessments are capable of detecting cancer cases with atypical characteristics, their efficiency and reliability in various clinical contexts need to be validated through extensive studies. Notably, validation of AI in similar retrospective studies could be improved even further by incorporating synchronized and randomized datasets from multiple sites alongside conventional methods. This would provide more evaluation data, leading to a more accurate assessment of the model’s real-world effectiveness in clinical settings.

## Conclusion

Integrating AI into predictive oncology medicine clinical workflows promises to streamline processes and significantly improve efficiency (Figure 1). It facilitates convenient data sharing, allowing communication between specialists even if they are located remotely. This integration also aims to reduce test execution times and generate precise and highly reproducible results, thus minimizing interpatient variability. Such advances are critical to ushering in an era of prevention in which the focus shifts from the heavy counting of cancer deaths to the promising active accounting of risk and its management. This early and proactive approach, based on the management of the potential patient with cancer, represents a new paradigm shift in how we respond to cancer as an existing disease and as a challenge to maintain health status.

By contrast, however, to achieve the goal it is necessary to correctly become aware of the technological challenge that AI presents. The challenge lies not only in selecting the appropriate type of predictive model but also, more importantly, in its validation and practical implementation. The validation process of the predictive model is the final key that determines the clinical potential of the model. Validating a predictive model primarily involves demonstrating that the predictions it makes genuinely affect outcomes, or in other words, that the risk percentages it provides for developing cancer are accurate and meaningful. Furthermore, model implementation must be carefully managed to address a variety of risks related to patient safety, data integrity, legal and ethical compliance, and technical challenges. These aspects have been discussed in several reports (Table 1) and, in particular, in the reflection paper (part of the European Medicines Agency’s initiatives) on the use of AI.^16^ The paper promotes validation by comparing the results of AI with independent analyses directly supervised by humans: this is essential for the transition from disconnected studies to reliable studies. In this direction, the validation of the predictive models adopted in retrospective studies contributes substantially.^17^ However, a limitation of these studies is that they rely on archival radiological imaging material. Radiological tests cannot be considered noninvasive or constantly repeatable over time. Consequently, the development of retrospective evaluation models based on data acquired in real-time or synchronously (i.e. LB) would be useful to improve the development of predictive models. This approach is not unknown in the world of AI (it has been applied, remarkably, in the paleontological field): however, there are currently no published studies in the specific field of precision preventive medicine.

Considering the rigorous reliability standards for AI-based secondary prevention prediction studies, it is crucial to rely on large, multicentre retrospective studies validated using external datasets. Furthermore, these studies could benefit from ‘stress mode’ testing, where input data are deliberately designed to challenge the model’s predictive capabilities. In the latest research where AI is applied to primary prevention and the predictive model is linked to a specific analytical model, the validation process should include both individual and combined tests. These tests are essential to evaluate the two aspects of the model, namely, the analytical component and the algorithm, through progressively more complex scenarios.
